# Supplementary material for: Revealing trends and persistent cycles of non-autonomous systems with autonomous operator-theoretic techniques
Source: Nat Commun. 2024 May 20;15:4268. doi: 10.1038/s41467-024-48033-6 (PMC11106270; doi:10.1038/s41467-024-48033-6)
Supplement: Supplementary file 1 — Supplementary Information [file 41467_2024_48033_MOESM1_ESM.pdf]

Revealing trends and persistent cycles of non-autonomous systems with autonomous operator-theoretic techniques

Gary Froyland[1,*], **Dimitrios Giannakis**[2,3], **Edoardo Luna**[4], **and Joanna Slawinska**[2]

[1]School of Mathematics and Statistics, University of New South Wales, Sydney, NSW 2052, Australia
[2]Department of Mathematics, Dartmouth College, Hanover, NH 03755, USA
[3]Department of Physics and Astronomy, Dartmouth College, Hanover, NH 03755, USA
[4]Department of Physics, University of Texas at Austin, Austin, TX 78712, USA
[*]g.froyland@unsw.edu.au

SUPPLEMENTARY INFORMATION

The contents of the Supplementary Information are as follows:

- Eigenvalue spectrum of the generator extracted from Indo-Pacific SST (Supplementary Fig. 1 and Supplementary Table 2).
- SST trend from transfer operator calculations (Supplementary Note 1 and Supplementary Fig. 2).
- δ^{18}O trend and frequency analysis from transfer operator calculations (Supplementary Note 2 and Supplementary Fig. 3).
- δ^{18}O trend and frequency analysis from Singular Spectrum Analysis (SSA) calculations (Supplementary Note 3 and Supplementary Fig. 4).
- Reconstruction of the δ^{18}O time series using generator eigenfunctions (Supplementary Note 4; Supplementary Fig. 5).
- Details of data and operator parameters for SST calculations (Supplementary Table 1).
- Details of data and operator parameters for δ^{18}O calculations (Supplementary Table 3).

Supplementary Note 1. SST trend from transfer operator calculations

We construct the transfer operator for the Indo-Pacific SST analysis as described in the "Indo-Pacific SST calculations" section in Methods. As per Supplementary Table 1, we use a single lag of 12 months, and step forward 6 months at a time. Supplementary Fig. 2 (left) displays the leading nontrivial real eigenvector – corresponding to eigenvalue $\lambda_2 = 0.4135$ – in blue, and global SST in green. Similarly, Supplementary Fig. 2 (right) compares the same eigenvector with global SAT.

In both cases, the warming trend extracted by the transfer operator from Indo-Pacific SST fields closely matches the global SST and SAT time series.

Supplementary Note 2. δ^{18}O analysis using transfer operators

We construct the transfer operator for the δ^{18}O analysis as described in the "Benthic δ^{18}O calculations" section in Methods. Supplementary Fig. 3 (panel (b)) displays the second eigenvector of the transfer operator approximation (corresponding to eigenvalue $\lambda_2 = 0.9632$), which is qualitatively consistent with the trend in the mean of the signal. Panels (c) and (d) of Supplementary Fig. 3 show the real parts of two distinct complex eigenvectors. These are the leading two complex-conjugate pairs, and have eigenvalues $\lambda_4 = 0.7639 + 0.3651i$ and $\lambda_6 = 0.3869 + 0.7216i$. From the arguments of these eigenvalues (divided by 7 because we are stepping forward 7 sampling intervals in one iteration of our transfer operator; see Supplementary Table 3) we obtain corresponding cycle periods of 98.64 and 40.78 kyr, which match the accepted distinct MPT cycle periods very well. Importantly, we note that the amplitude of the oscillations of the eigenvectors in the two rightmost panels of Supplementary Fig. 3 are largest in the time domain corresponding to when the oscillation is present. Panels (e) and (f) of Supplementary Fig. 3 display the complex time series of eigenvector values from panels (c) and (d) respectively, where the coloring is according to time; this further illustrates the concentration of large amplitudes in late (resp. early) temporal epochs. Continuous wavelet transforms (CWT) of panels (a)–(d) are shown in panels (g)–(j).

In summary, the eigenvectors of the transfer operator have separated the trend in the mean of the δ^{18}O time series from the oscillatory components. Further, the complex eigenvectors have separated and identified the two dominant frequencies, including indicating when each frequency is present through the amplitude of the eigenvectors. The results in the first row of Fig. 10 are also consistent with Supplementary Fig. 3.

Supplementary Note 3. δ^{18}O analysis using SSA

In Supplementary Fig. 4, we show results obtained from SSA applied to the same δ^{18}O dataset as in our Koopman/transfer operator computations, using the same delay embedding parameters as in the computation of generator eigenfunctions (i.e., $Q = 150$, $\ell = 1$; see Supplementary Table 3). The leading mode in the SSA spectrum (shown in Supplementary Fig. 4(b)) exhibits trend-like behavior similar to generator eigenvector v_2 in Fig. 10(b), but it is significantly noisier and appears to mix secular evolution with oscillatory signals (see the CWT spectrum in Supplementary Fig. 4(k)). The next modes in the SSA spectrum consist of two oscillatory pairs, $\{v_2^{\mathrm{SSA}}, v_3^{\mathrm{SSA}}\}$ and $\{v_4^{\mathrm{SSA}}, v_5^{\mathrm{SSA}}\}$, with periodicities close to the 100 kyr and 41 kyr periodicities of glaciation cycles after and before the MPT, respectively (see Supplementary Fig. 4(c, d, g, h, l, m). However, compared to their generator eigenfunction counterparts, $\{v_6, v_7\}$ and $\{v_4, v_5\}$, respectively, the oscillatory SSA modes exhibit significantly weaker amplitude modulation in the periods before and after the MPT, and thus do not provide a clear delineation of the transition. We note that there exist modifications of the standard SSA algorithm that perform rotations of the eigenvectors to produce modes that are more skillful at separating trends and distinct oscillatory components[1,2]. Application of these methodologies is outside the scope of the present work, but they could potentially lead to improvements over the results depicted in Supplementary Fig. 4.

Supplementary Note 4. Reconstruction of δ^{18}O time series

To explore the evolution of glaciation cycles as revealed by the eigenfunctions from Fig.10, in Supplementary Fig. 5 we compare the raw δ^{18}O time series (Fig. 10(a)) to reconstructed δ^{18}O time series computed using the reconstruction procedure described in Methods. We start from a reconstruction based on eigenfunction v_2 in Fig. 10(b), and then progressively add contributions from pairs of oscillatory eigenfunctions in Panels (b–e). As expected, the reconstruction based on v_2 alone exhibits a secular evolution that closely

resembles the v_2 time series. In Supplementary Fig. 5(c), we see that adding oscillatory pair $\{v_6, v_7\}$ produces a 100 kyr cycle in the post-MPT period with minimal modification of the pre-MPT evolution. In Supplementary Fig. 5(d), adding pair $\{v_4, v_5\}$ generates a 40 kyr cycle that is primarily concentrated in the pre-MPT period and a 40 kyr to 100 kyr switch around 1 Ma. Finally, adding pair $\{v_9, v_{10}\}$ (the secondary 41 kyr oscillation) modifies the signal over the full 3 Myr analysis interval, and visibly contributes to the irregularity of post-MPT glaciation cycles. We therefore see that with a modest number of eigenfunctions our approach recovers a "skeleton" of the evolution of NH Quaternary glaciation cycles that captures its salient observed features.

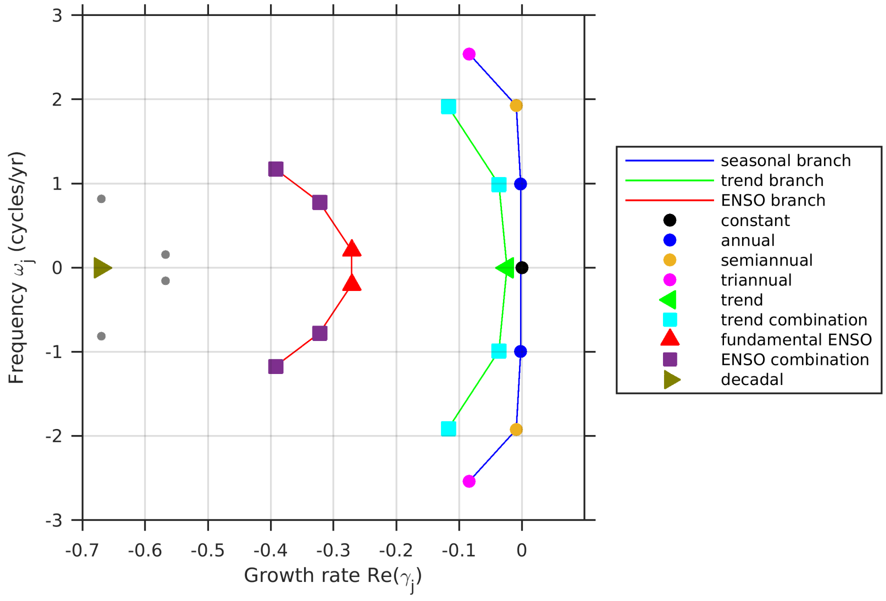

Supplementary Figure 1. Leading generator eigenvalues γ_j computed from monthly-averaged Indo-Pacific SST data from ERSSTv4 over the period January 1891 to December 2019. The vertical and horizontal axes show the frequency $\omega_j = \mathrm{Im}(\gamma_j)/(2\pi)$ and growth rate, $\mathrm{Re}(\gamma_j)$, respectively. Note that complex eigenvalues occur in complex-conjugate pairs as appropriate for describing oscillatory signals at the corresponding eigenfrequencies. Moreover, negative values of $\mathrm{Re}(\gamma_j)$ correspond to decay. Following Fig. 3 in ref.[3] – which used Indo-Pacific SST time series from January 1970 to February 2020 – we group the eigenvalues into branches associated with seasonal (periodic), trend, ENSO, and decadal eigenfunctions. Lines connecting eigenvalues serve as visual guides for these spectral branches. A listing of the parameters employed for generator approximation can be found in Supplementary Table 1.

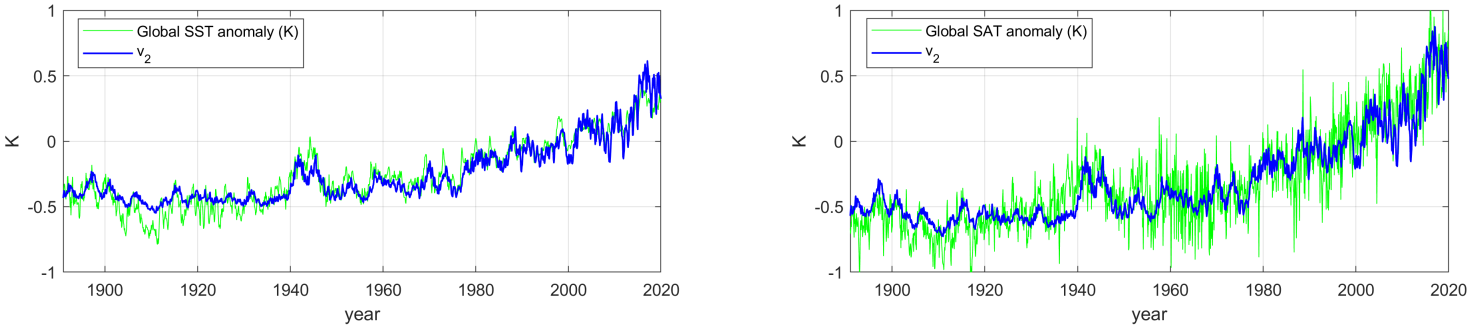

Supplementary Figure 2. Comparison of leading transfer operator eigenvector with global SST and SAT anomalies. *Left:* The second eigenvector of the transfer operator (the first nontrivial real eigenvector) shown in blue captures the trend in the Indo-Pacific SST signal. The globally averaged SST anomaly is shown in green. *Right:* The same second eigenvector is shown in blue. The globally averaged SAT anomaly is shown in green. In each case, the eigenvector has been affinely scaled to convert its arbitrary units to Kelvin.

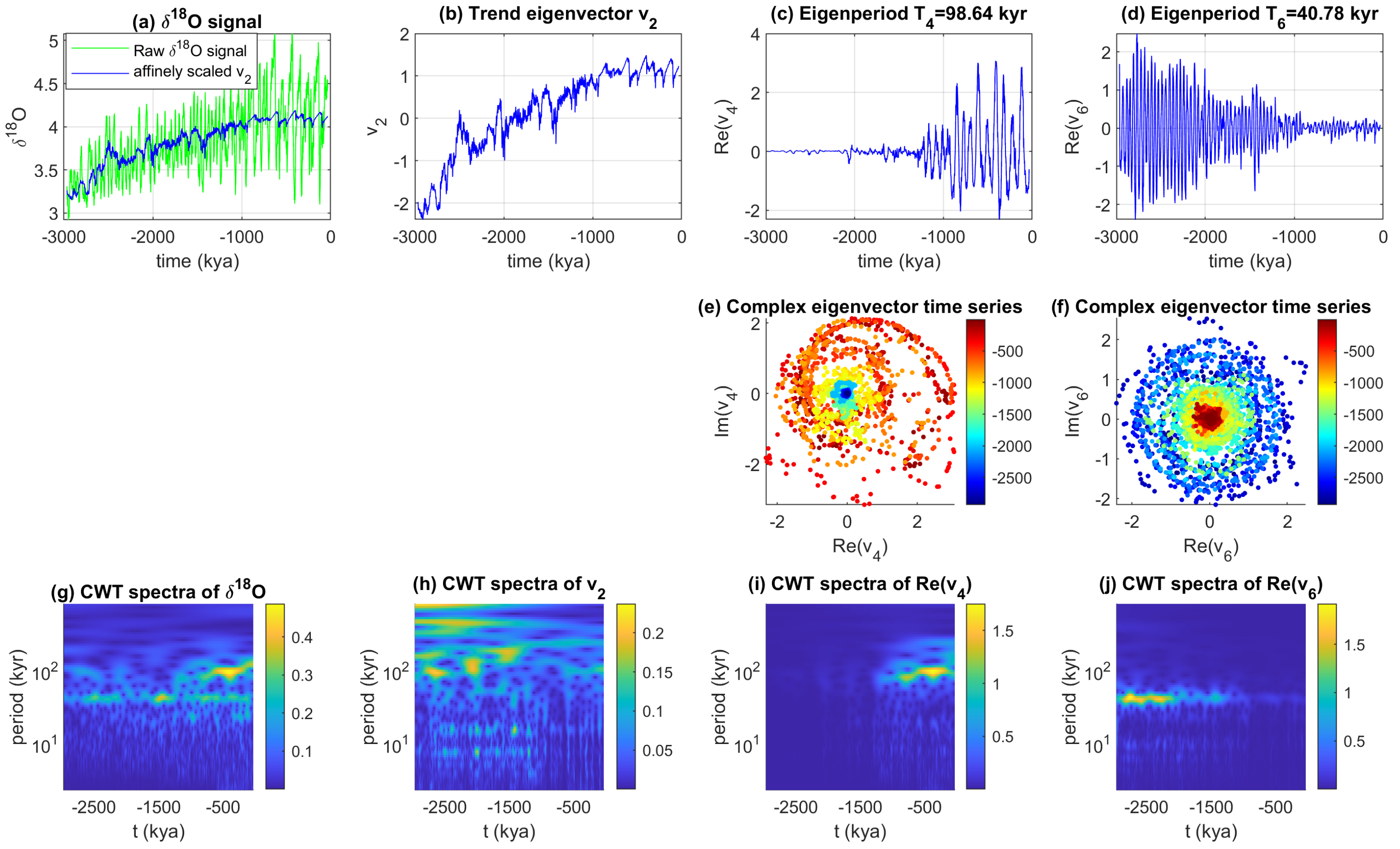

Supplementary Figure 3. Transfer operator analysis of δ^{18}O data from the LR04 stack over the past 3 Myr. Panel (a) shows the raw δ^{18}O time series over the past 3 Myr (green) with affinely scaled transfer operator eigenfunction v_2 (blue). The second eigenfunction v_2 (the first nontrivial real eigenfunction) captures the drift in the mean of the raw δ^{18}O signal. The eigenfunction has been affinely scaled to convert its arbitrary units to δ^{18}O isotope concentration ratio. Panel (b) displays the time series of transfer operator eigenfunction v_2 representing trend. Panel (c) shows the time series of the real part of the complex eigenfunction v_4 (from the first nontrivial complex eigenfunction pair), displaying an oscillation period of 98,640 yr, which corresponds to oscillations in the δ^{18}O time series over the most recent 1.5 million years. Panel (d) displays the time series of the real part of the complex eigenfunction v_6 (from the second nontrivial complex eigenfunction pair), displaying an oscillation period of 40,775 yr, which corresponds to oscillations in the δ^{18}O time series from 3 Ma to 2 Ma ago. Panels (e–f) illustrate two-dimensional phase space plots of v_4 and v_6 in the complex plane, colored by time. Panels (g–j) show CWT spectra of the δ^{18}O time series (g), and the CWT spectra of the time series of transfer operator eigenfunctions v_2, v_4, and v_6 (h–j).

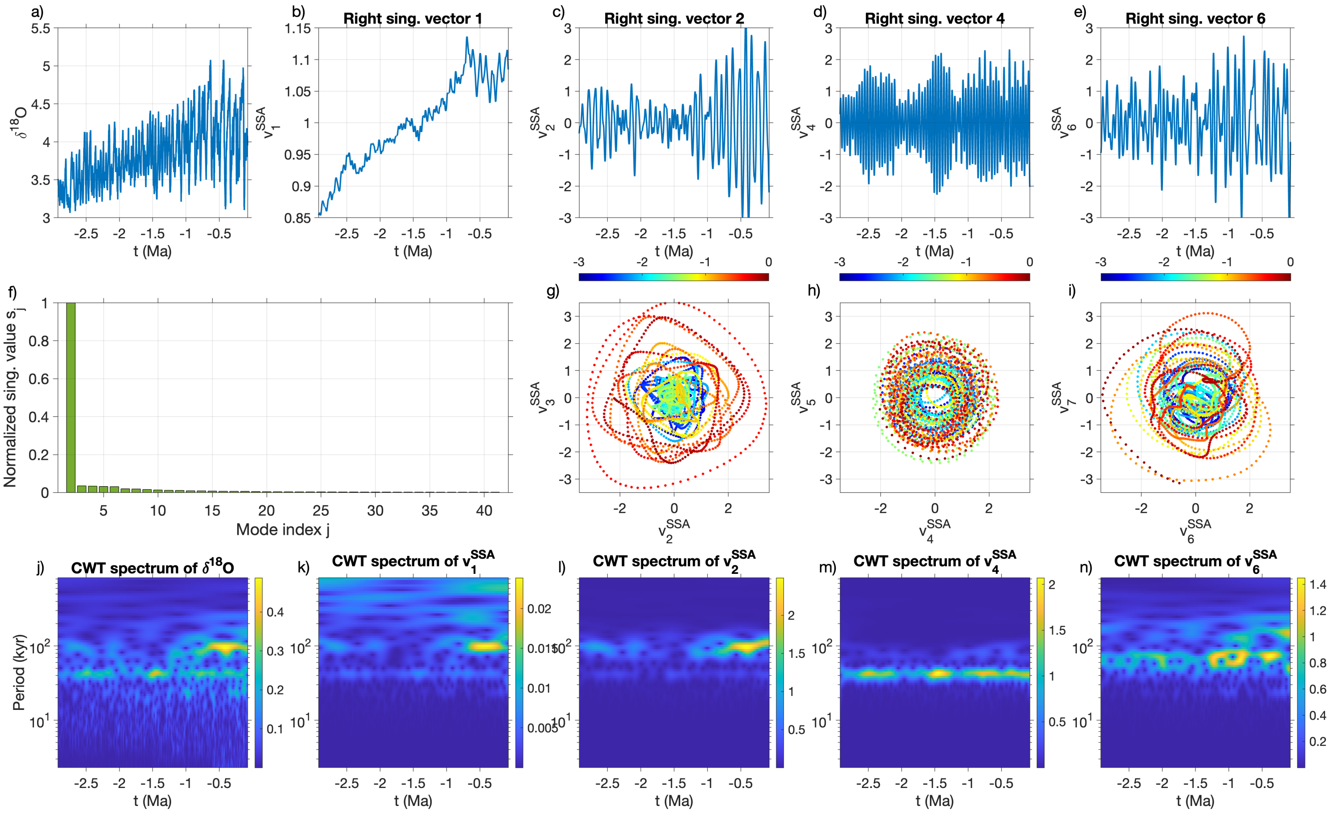

Supplementary Figure 4. **Leading right singular vectors (temporal modes) from SSA applied to the δ^{18}O data.** (a) Raw δ^{18}O time series over the past 3 Myr. (b) Time series of the leading SSA temporal mode v_1^{SSA} representing trend. (c–e) Time series of oscillatory modes v_4^{SSA}, v_6^{SSA}, and v_8^{SSA}, respectively. (f) Normalized singular values $s_j = \sigma_j/\sigma_1$ of the leading 41 SSA modes. Here, $\sigma_1, \sigma_2, \ldots$ are the singular values of the SSA covariance operator, ordered in decreasing order. (g–i) Two-dimensional phase space plots of oscillatory pairs $\{v_2^{\mathrm{SSA}}, v_3^{\mathrm{SSA}}\}$, $\{v_4^{\mathrm{SSA}}, v_5^{\mathrm{SSA}}\}$, and $\{v_6^{\mathrm{SSA}}, v_7^{\mathrm{SSA}}\}$, respectively, colored by time. (j–n) CWT spectra of the δ^{18}O time series (j) and the time series of modes v_1^{SSA}, v_2^{SSA}, v_4^{SSA}, and v_6^{SSA} (k–n).

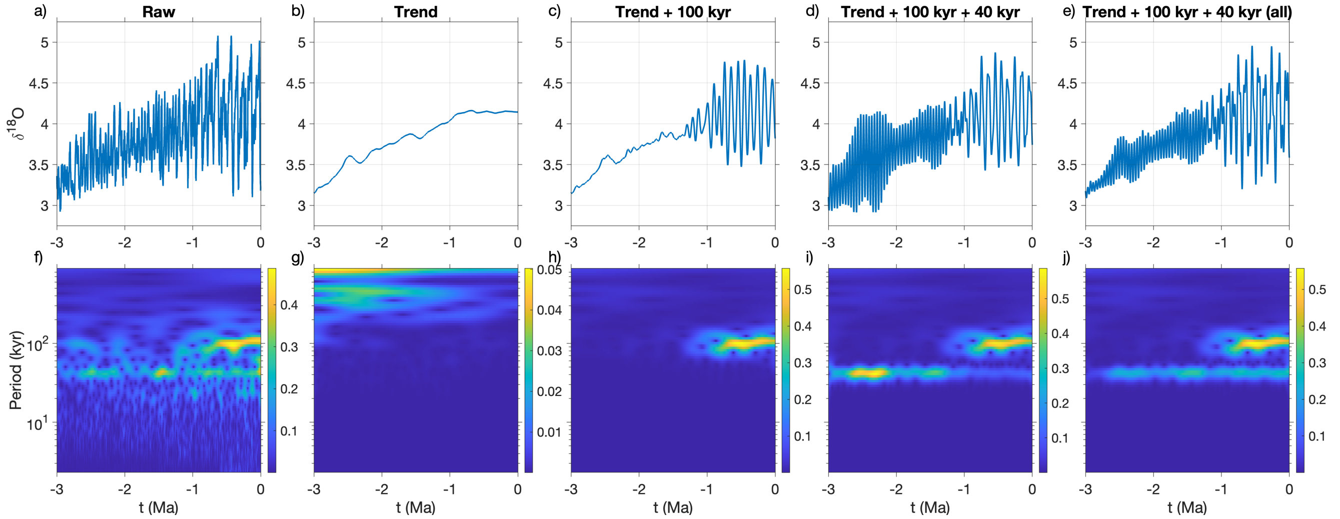

Supplementary Figure 5. Time series of reconstructed δ^{18}O using various combinations of eigenfunctions depicted in Fig. 10. Panels (a, f) show the raw δ^{18}O time series and its CWT spectrum similarly to Panels (a, j) of Fig. 10. Panels (b–e) show δ^{18}O reconstructions based on: (b) trend eigenfunction v_2, (c) v_2 and the dominant 100 kyr oscillatory pair $\{v_6, v_7\}$; (d) v_2, v_6, v_7, and the dominant 41 kyr oscillatory pair $\{v_4, v_5\}$; and (e) v_2, v_6, v_7, v_4, v_5 and the secondary 41 kyr oscillatory pair $\{v_9, v_{10}\}$. Panels (g–j) show the CWT spectra of the reconstructed time series in Panels (b–e), respectively.

Supplementary Table 1. Dataset attributes and numerical parameter values for the analyses of Indo-Pacific SST from ERSSTv4. See Methods in ref.[3] for further details on the generator approximation parameters.

Dataset Attributes	
Analysis date range	Jan 1891 – Dec 2019
Climatology date range[a]	Jan 1971 – Dec 2000
Sampling interval Δt	1 month
Number of snapshots N	1548
SST analysis domain	28°E–70°W, 60°S–20°N
Nominal resolution	2°
Number of gridpoints d	4868
Generator Approximation	
Number of delays Q	48
Timesteps between delays ℓ	1
Kernel bandwidth parameter γ	33
Cone kernel parameter ζ	0.995
Number of kernel eigenfunctions L	400
Generator regularization parameter ε	0.001
Transfer Operator Approximation	
Number of delays Q	2
Timesteps between delays ℓ	12
Number of forward steps s	6
Number of nearest neighbors K	5

[a]Used to compute SST and SAT anomalies.

Supplementary Table 2. Eigenfrequencies and eigenperiods corresponding to the leading 25 generator eigenfunctions extracted from Indo-Pacific ERSSTv4 data. See Methods in ref.[3] for further details on the generator approximation parameters.

	Frequency (yr^{-1})	Period (yr)	Type
v_1	0.000	∞	constant
v_2	0.995	1.005	annual
v_3	−0.995	−1.005	annual
v_4	1.925	0.520	semiannual
v_5	−1.925	−0.520	semiannual
v_6	0.000	∞	trend
v_7	0.988	1.012	trend combination
v_8	−0.988	−1.012	trend combination
v_9	2.539	0.394	triannual
v_{10}	−2.539	−0.394	triannual
v_{11}	1.913	0.523	trend combination
v_{12}	−1.913	−0.523	trend combination
v_{13}	0.204	4.910	ENSO
v_{14}	−0.204	−4.910	ENSO
v_{15}	0.779	1.284	ENSO combination
v_{16}	−0.779	−1.284	ENSO combination
v_{17}	1.172	0.853	ENSO combination
v_{18}	−1.172	−0.853	ENSO combination
v_{19}	0.157	6.386	
v_{20}	−0.157	−6.386	
v_{21}	0.817	1.225	
v_{22}	−0.817	−1.225	
v_{23}	0.000	∞	decadal
v_{24}	1.727	0.579	
v_{25}	−1.727	−0.579	

Supplementary Table 3. Dataset attributes and numerical parameter values for the analyses of δ^{18}O concentration data from the LR04 stack.

Dataset Attributes	
Analysis date range	3 Ma – present
Sampling interval Δt	1 kyr
Number of snapshots N	3001
Generator approximation	
Number of lags Q	150
Timesteps between lags ℓ	1
Kernel bandwidth parameter γ	0.216
Cone kernel parameter ζ	0
Number of kernel eigenfunctions L	81
Generator regularization parameter ε	0.001
Transfer operator approximation	
Number of delays Q	5
Timesteps between delays ℓ	15
Number of forward steps s	7
Number of nearest neighbors K	7

Supplementary References

1. Groth, A. & Ghil, M. Multivariate singular spectrum analysis and the road to phase synchronization. *Phys. Rev. E* **84**, 036206, DOI: 10.1103/PhysRevE.84.036206 (2011).

2. Groth, A. & Ghil, M. Monte carlo singular spectrum analysis (SSA) revisited: Detecting oscillator clusters in multivariate datasets. *J. Clim.* **28**, 7873–7893, DOI: 10.1175/JCLI-D-15-0100.1 (2015).

3. Froyland, G., Giannakis, D., Lintner, B., Pike, M. & Slawinska, J. Spectral analysis of climate dynamics with operator-theoretic approaches. *Nat. Commun.* **12**, 6570, DOI: 10.1038/s41467-021-26357-x (2021).
